# Structure, Function, and Interactions of the HIV-1 Capsid Protein

**DOI:** 10.3390/life11020100

**Published:** 2021-01-29

**Authors:** Eric Rossi, Megan E. Meuser, Camille J. Cunanan, Simon Cocklin

**Affiliations:** 1 Angle North America, 1500 1st Avenue, Suite 1010, King of Prussia, PA 19462, USA; e.rossi@angleplc.com; 2 Department of Biochemistry & Molecular Biology, Drexel University College of Medicine, Rooms 10307, 10309, and 10315, 245 North 15th Street, Philadelphia, PA 19102, USA; mem484@drexel.edu (M.E.M.); cjc435@drexel.edu (C.J.C.)

**Keywords:** HIV-1/AIDS, capsid, host proteins, post-entry events, assembly, virus-host interactions, restriction factors

## Abstract

The capsid (CA) protein of the human immunodeficiency virus type 1 (HIV-1) is an essential structural component of a virion and facilitates many crucial life cycle steps through interactions with host cell factors. Capsid shields the reverse transcription complex from restriction factors while it enables trafficking to the nucleus by hijacking various adaptor proteins, such as FEZ1 and BICD2. In addition, the capsid facilitates the import and localization of the viral complex in the nucleus through interaction with NUP153, NUP358, TNPO3, and CPSF-6. In the later stages of the HIV-1 life cycle, CA plays an essential role in the maturation step as a constituent of the Gag polyprotein. In the final phase of maturation, Gag is cleaved, and CA is released, allowing for the assembly of CA into a fullerene cone, known as the capsid core. The fullerene cone consists of ~250 CA hexamers and 12 CA pentamers and encloses the viral genome and other essential viral proteins for the next round of infection. As research continues to elucidate the role of CA in the HIV-1 life cycle and the importance of the capsid protein becomes more apparent, CA displays potential as a therapeutic target for the development of HIV-1 inhibitors.

## 1. Introduction

**Figure 1:**
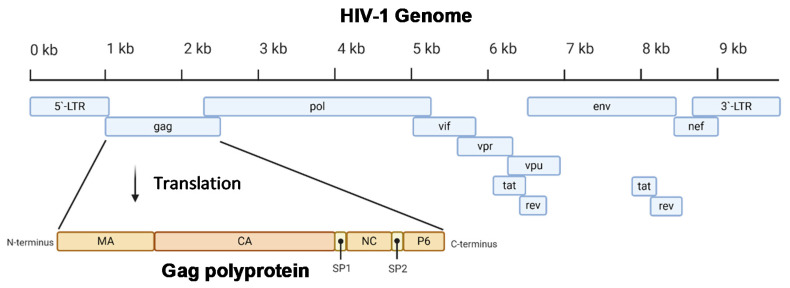
A diagram of the 9.8 kb HIV-1 genome. The Gag portion of the genome is transcribed into the Gag polyprotein, consisting of the matrix (MA), capsid (CA), nucleocapsid (NC), P6, and two spacer peptides (SP1 and SP2). During maturation, the polyprotein is cleaved into its constituent parts. *Image created with BioRender.com*.

**Figure 2:**
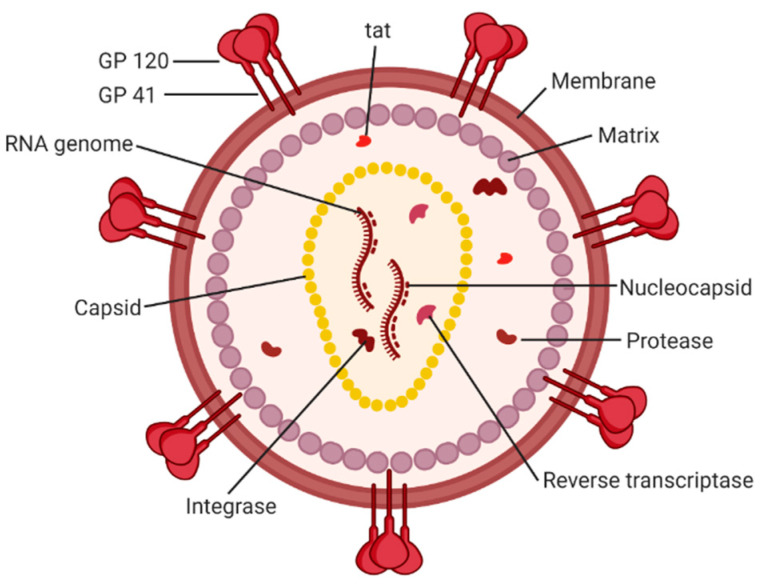
A schematic of the HIV-1 virion. Envelope proteins, GP41 and GP120, surround the host-derived membrane surface, which is lined internally with a layer of matrix protein. Inside the virion are viral proteins and the CA core containing the HIV-1 genome and proteins essential for infection. *Image created with BioRender.com*.

Acquired immunodeficiency syndrome (AIDS) affected approximately 38 million people in 2019. The etiologic agent for AIDS is the human immunodeficiency virus (HIV) [1]. While HIV is categorized into two subgroups, type 1 and type 2, HIV type 1 (HIV-1) is the most prevalent cause of AIDS worldwide [2]. It is an enveloped virus containing a 9.8kb positive-sense RNA genome (Figure 1) that codes for three polyproteins (Gag, Pol, and Env) and six accessory proteins (Tat, Rev, Nef, Vpr, Vif, and Vpu) [3]. HIV-1 targets human cells presenting the CD4 receptor and CCR5 or CXCR4 co-receptors, such as T helper cells and microglial cells [4,5]. It penetrates the host cell through receptor-mediated entry, which results in the viral core entering the cytoplasm of the host cell [6]. The capsid core is a fullerene-like cone made of the capsid (CA) portion of the Gag polyprotein. The core contains the viral genome and viral proteins essential for replication, such as integrase and reverse transcriptase (Figure 2) [7]. The CA protein is essential in both the early and late stages of the HIV-1 life cycle, with many host cell factors currently identified as direct binding partners [7].

**Figure 3:**
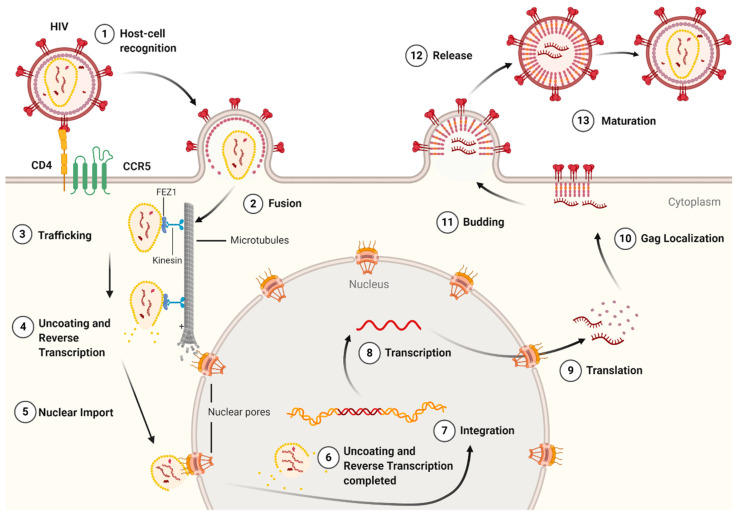
The life cycle of the HIV-1 virus. The early-stage begins with the recognition of host cell receptors (**1**), resulting in the fusion of the virus and release of the viral core into the cytoplasm of the host cell (**2**). This is followed by the trafficking of the core through the cytoplasm (**3**) as reverse transcription and uncoating begins to take place (**4**). Once at the nuclear pore, the viral contents are imported into the nucleus and localized (**5**) to transcriptionally active chromatin while uncoating and reverse transcription are completed (**6**). Following uncoating and reverse transcription, integration occurs (**7**). After the viral genome is integrated into the host cell, viral genes are transcribed (**8**) and translated (**9**) into the Gag polyprotein. The Gag polyprotein then localizes to the host cell membrane (**10**), where budding occurs (**11**), followed by the release of an immature virion (**12**). The final step in the HIV-1 lifecycle is maturation (**13**), where the viral protease cleaves the Gag polyprotein into its constituent, functional proteins. *Image created with BioRender.com*.

The life cycle of HIV-1 (Figure 3) can be broken down into two stages: early and late. The early-stage begins with an infectious virion binding to the host cell and ends with the integration of the viral genome into the host DNA. The late stage of the life cycle is the period from post-integration until viral maturation [8]. The HIV-1 life cycle’s early stage begins with the virion’s glycoprotein complex, Env, interacting with the CD4 receptor and the CCR5 or CXCR4 co-receptors on the host cell [9]. This recognition event initiates a cascade of conformational rearrangements that results in viral fusion, where the viral core is released into the cytoplasm of the host cell [9]. The complex of the capsid protein and its contents is referred to as the reverse-transcription complex (RTC) [7]. From here, reverse transcription must occur within the core. During reverse transcription, capsid begins uncoating and trafficking to the nucleus for import and integration [10]. Once the RTC has entered the nucleus, it is referred to as the pre-integration complex (PIC) [7]. The processes of uncoating and reverse transcription are then completed after nuclear import and before nuclear integration [11,12,13]. The capsid is responsible for localizing the PIC to transcriptionally active sites on chromatin and facilitating the integration of the viral transcripts into the host genome [11,14,15,16]. The viral genes are then transcribed by the host cell and exported from the nucleus. Export is followed by localization of the Gag protein to the plasma membrane through a myristoyl group at the amino-terminus of Gag [17]. Localization is followed by an immature virion budding, with intact Gag polyproteins coating the host-derived viral membrane [18]. The final step of the viral life cycle is maturation, which results in a fully infectious virion. The maturation step is facilitated by the viral protease that cleaves the polyprotein into its smaller, functional constituents [19]. In this step, the viral capsid is formed and assembles into the fullerene-cone, forming a mature and fully infectious HIV-1 virion. At this stage, the newly formed and matured virion can restart the HIV-1 life cycle, infecting another host cell [19].

Continued studies into the life cycle of HIV-1 have revealed that CA is essential in infection and replication. It provides structure and protection to the RTC and PIC while allowing dNTP diffusion for reverse transcription [20,21]. Additionally, it facilitates retrograde movement in the cytoplasm, nuclear import, and localization [12,14,22,23,24,25,26,27]. Since CA is essential in the HIV-1 lifecycle, it is a promising target for future research into HIV-1 inhibitors [28,29,30,31,32,33,34].

## 2. Structure of the HIV-1 Capsid

### 2.1. Gag Polyprotein

The proteins of HIV-1 are transcribed and translated as polyproteins, which are long peptides that are cleaved into smaller, functional units by the viral protease [35]. The CA protein of HIV-1 is translated as a constituent of the larger Gag polyprotein (Figure 1) that contains many integral viral components [36]. This intact Gag protein is essential in the late stages of the viral life cycle. It is responsible for the localization of viral components to the host cell plasma membrane through a myristoyl group located at its amino-terminus [17]. Following budding, the virus is still immature because the Gag polyprotein remains whole. It is not until cleavage by the viral protease that the polyprotein is separated into its individual constituents, and a mature, infectious virion is formed [37].

### 2.2. CA Monomer: The Building Block of the Capsid Core

**Figure 4:**
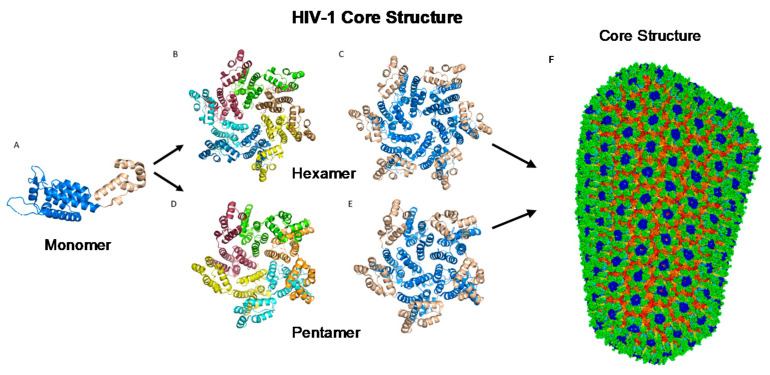
HIV-1 CA monomers oligomerize into hexamers and pentamers that assemble to form the capsid fullerene-cone core. (**A**) Shows the CA monomer with the amino-terminus colored blue and the carboxyl-terminus colored tan (PDB 3H47). (**B**) Shows the CA hexamer with each subunit colored differently (PDB 3H47). (**C**) Shows the hexamer with the amino-terminus of each subunit colored blue and the carboxyl terminus colored tan (PDB 3H47). (**D**) Shows the CA pentamer with each subunit colored differently (PDB 3P05). (**E**) Shows the pentamer with the amino-terminus of each subunit colored blue and the carboxyl terminus colored tan (PDB 3P05). (**F**) The complete capsid core structure containing approximately 250 hexamer oligomers and 12 pentamer oligomers (PDB 3J3Q). Image created with the PyMOL Molecular Graphics System, Version 2.4 Schrödinger, LLC.

The most basic component of the capsid core is the CA monomer (Figure 4A), a highly conserved and genetically fragile protein [38]. The CA monomer is a 231-residue long protein with two major alpha-helical domains, the N-terminal and C-terminal domains (CA-NTD and CA-CTD, respectively) that are connected by a five-residue linker (residues 146–150) [39]. While a virion is in its immature form, CA is a constituent of the Gag polyprotein, linked to the matrix (MA) domain at its N-terminus and the spacer peptide 1 (SP1) domain of the polyprotein at its C-terminus (Figure 1). After budding and during maturation, the Gag polyprotein undergoes proteolytic cleavage, separating it from the MA and SP1 domains. Interactions then occur between CA residues Ala14 and Glu45 of adjacent monomers, allowing the monomers to form larger oligomers (Figure 7) [40]. One study that examined CA dynamics determined that the protein exists in an equilibrium of monomers and dimers. In this equilibrium, the orientation of the NTD and CTD exists in a range of different states. This equilibrium was suggested to account for the pentamer/hexamer configuration seen in the fully assembled CA core, with the variability of the NTD of the dimer determining the pentamer/hexamer pattern of assembly in the finished core [41].

### 2.3. CA Oligomers: Pentamers and Hexamers

**Figure 5:**
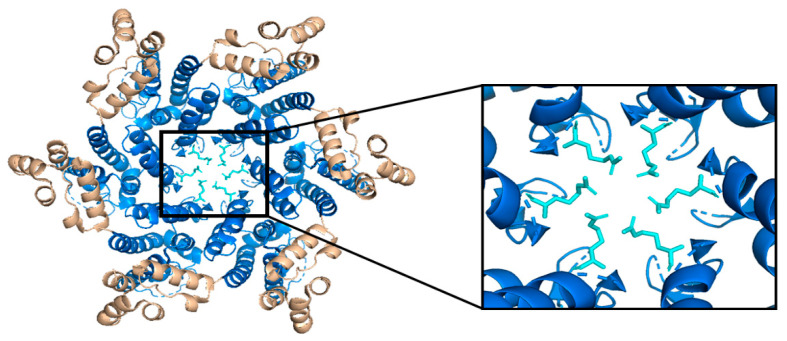
The HIV-1 CA hexamer with the R18 ring expanded. The amino-terminus is colored blue, the carboxyl-terminus is colored tan, and each subunit’s R18 residue is colored in cyan. This is referred to as the R18 ring. This pore allows for the diffusion of materials needed for reverse transcription while sequestering reverse transcription machinery from host cell restriction factors. (PDB 3H47) Image created with the PyMOL Molecular Graphics System, Version 2.4 Schrödinger, LLC.

While the HIV-1 CA monomers may form various curved oligomers, the most abundant oligomer in the mature cone is the hexamer (Figure 4B,C). Approximately 250 hexamers and precisely 12 pentamers (Figure 4D,E) form the fullerene-like cone lattice of the viral capsid core (Figure 4F) [40]. The hexamers are structured with the N-terminal domain forming a central, stable core and the C-terminal domains forming a flexible outer ring [40]. The center of the hexamer core, referred to as the R18 pore, is lined with six arginine residues and a “molecular-iris” formed from the N-terminal β-hairpin (Figure 5). This pore is a size-selective, positively charged channel that allows for the diffusion of nucleotides into the capsid core while preventing access to nucleases, host cell restriction factors, and sensors [20].

**Figure 6:**
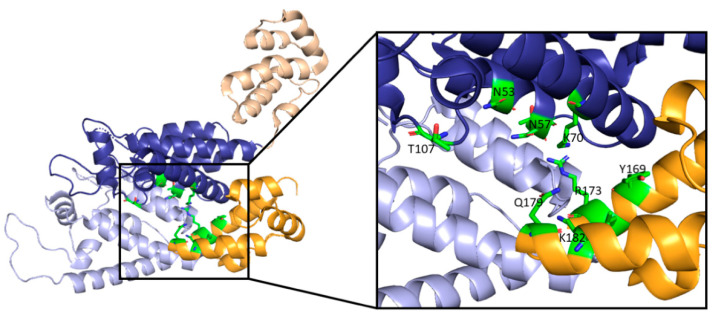
Here, two adjacent CA protomers of the CA hexamer form a functional interprotomer pocket. N-terminal domains (NTDs) are colored in shades of blue, and C-terminal domains (CTDs) are colored in shades of orange. Interacting domains are marked in dark blue and bright orange. Interacting residues within the interaction interface are magnified and colored in green. (PDB 6MQA) Image created with the PyMOL Molecular Graphics System, Version 2.4 Schrödinger, LLC.

Along with the R18 pore, there is a crucial interprotomer pocket (Figure 6) that forms between hexameric subunits and is generated by the NTD of one subunit interacting with the CTD of another subunit. This NTD-CTD interprotomer interface is present in the mature HIV-1 capsid and is critical for proper capsid assembly, stability, and host cell factor recognition. There is evidence that this pocket binds host cell factors involved in nuclear import, suggesting that intact CA oligomers are imported into the nucleus during nuclear entry of the pre-integration complex (PIC) [42]. Due to these roles, the interprotomer interface has been used as a target for small-molecule inhibition [43].

Additional interactions involved in the polymerization of capsid monomers include the NTD-NTD interactions that promote the formation of hexamers and CTD-CTD interfaces that connects hexameric units into the conical lattice [40,44]. The NTD-NTD interface involves helices 1 and 3 of one monomer and helix 2 of the adjacent monomer [40]. Conversely, the CTD-CTD interface links two neighboring capsid subunits belonging to different hexamers and is defined by helix 9 of the adjacent monomers [44].

### 2.4. The Fullerene Cone

**Figure 7:**
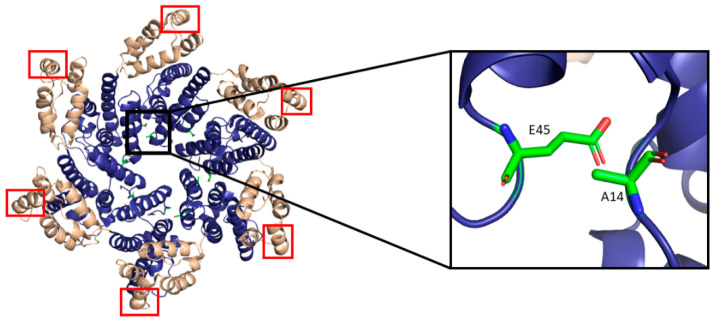
The HIV-1 CA hexamer with regions important for higher-order oligomeric assembly are highlighted. The amino-terminus is colored blue, and the carboxyl-terminus is colored tan. Residues A14 and E45 between monomeric subunits are magnified and colored green. The regions of hydrophobic residues within the three-helix bundle center are marked with red boxes. (PDB 6MQA) Image created with the PyMOL Molecular Graphics System, Version 2.4 Schrödinger, LLC.

The hexamers and pentamers associate into the curved lattice that forms the mature capsid core. Electron microscopy has demonstrated that the natural viral core exhibits cone angles of ~19°, indicating that the capsid core of HIV-1 is structured as a fullerene cone, with the rigid rotation between the pentamers and hexamers appearing to generate the continuous curve of the fullerene cone [45,46]. However, both perfect and aberrant fullerene cones have been observed after maturation and are believed to be caused by the de novo capsid assembly that occurs after proteolytic cleavage of the Gag polyprotein [47]. The fullerene cone is an important structure, as it protects the viral genome from a wide range of host cell factors that would otherwise restrict infection. Mutagenesis studies revealed that residues within the interprotomer pocket and pentamer/hexamer interface are essential, with disruption of those interactions leading to decreased capsid assembly, stability, and viral infectivity [48,49,50,51,52,53]. Specifically, the hydrophobic residues in the three-helix bundle center within the CA-CTD are crucial for hexamer/pentamer association (Figure 7) [37]. This again emphasizes the importance of a stable capsid core since instability in the core has been shown to cause a loss of the viral genome and integration [54].

## 3. Function of the HIV-1 Capsid

The HIV-1 capsid possesses many functions that are due to the interactions of capsid with various host cell factors. The capsid core is responsible for the safe delivery of the viral genome and reverse-transcription machinery from the periphery of the cytoplasm to transcriptionally active locations in the nucleus. It permits for this safe delivery by preventing restriction factors from sensing the viral genome while allowing the proper molecules to diffuse through the core to allow for reverse transcription to occur.

### 3.1. Nucleotide Diffusion through R18 Pore Is Accelerated by ATP and IP6 Interactions

One of the earliest known functions of the capsid core is to protect the reverse transcription machinery from host cell restriction factors that would prevent HIV-1 reverse transcription and proper infection. While the reverse transcription machinery is sequestered, an influx of dNTPs is needed to complete the reverse transcription process, accomplished through the R18 pore. Studies have determined that dNTPs can pass through the R18 pore by diffusion through the positive electrostatic field within the pore [20]. The potential of the R18 pore attracts dNTPs where they can bind, becoming confined. While confined in the ring, diffusion through the pore is favored over its movement back into the cytoplasm. In addition to dNTPs, it has been observed that inositol hexaphosphate (IP6) and ATP can also bind to the R18 pore, and the competition for binding between these molecules accelerates the rate of dNTP diffusion by accelerating the release of the bound dNTP [21]. It was calculated that the binding of ATP or additional dNTPs accelerates release through the pore approximately 10^3^-fold, while IP6 binding results in an increase in the release rate of approximately 10^6^-fold. This diffusion mechanism is sufficient to supply dNTPs for encapsidated reverse transcriptase [21].

### 3.2. Capsid Uncoating, Reverse Transcription, and Associated Host Factors

#### 3.2.1. Capsid Uncoating Is Essential for and Timed by Reverse Transcription

Following membrane fusion, the core is released into the cytoplasm of the host cell, where it must be uncoated and trafficked to the nucleus for nuclear import so that integration of the viral genome can occur. Upon entry into the cell, the viral core forms the RTC. The RTC, in which the capsid core plays an essential role, is responsible for generating a DNA copy of the viral RNA genome for integration [55]. Prior to integration, the capsid must undergo the process of uncoating, in which the fullerene core begins to disassemble, allowing for the integration of the viral cDNA transcript into the host genome. Multiple theories of uncoating have been developed. Previous findings have illustrated that uncoating begins immediately after entry into the cytoplasm and is completed before nuclear import. However, it has thereafter been suggested that uncoating does not begin until after certain stages of reverse transcription and is completed after viral integration in the nucleus [56,57]. One study determined that capsid uncoating occurs approximately 30 min post-fusion, and most, but not all, of the CA is found in the cytoplasm of the cell [56]. However, it has recently been shown that the rate-limiting step of uncoating is the initial breach of the fullerene cone lattice and that different stages of the uncoating process involve various host cell factors [57]. The interactions with these host cell factors and the cellular environments of different cell types regulate the timing of the rate-dependent and essential uncoating process [49,57,58].

More recent studies have demonstrated that the uncoating process occurs after reverse transcription has started and is completed after nuclear entry [12,59,60]. Such studies show that uncoating does not begin until after the reverse transcription process completes the first strand transfer and that the capsid core is necessary to protect the RTC/PIC from degradation within the cytoplasm [10,61]. Additionally, the capsid core is required to mediate docking at the nuclear pore, indicating that uncoating does not occur until after nuclear docking [61]. Recent results have shown that intact capsid cores are imported into the nucleus through mechanisms involving host cell factor cleavage and polyadenylation specificity factor 6 (CPSF6) and that reverse transcription is completed near the site of integration less than one and a half hours before viral uncoating is completed [11,12,13].

Furthermore, it was shown that many reverse transcription intermediates are enriched in nuclear fractions suggesting that a large part of reverse transcription occurs in the nucleus [11]. All of this points to uncoating and reverse transcription taking place later than previously thought and completing in the nucleus. The latest research revealed the formation of a single-stranded RNA molecule into a filamentous coiled structure through high-resolution mechanical mapping. This stiff structure was enough to increase the core’s internal pressure and then breach the capsid lattice, resulting in the commencement of uncoating [62]. This now strongly indicates that the capsid core undergoes nuclear import and that reverse transcription and uncoating are linked, and both are completed within the nucleus.

#### 3.2.2. CypA Stabilizes the Capsid Core

**Figure 8:**
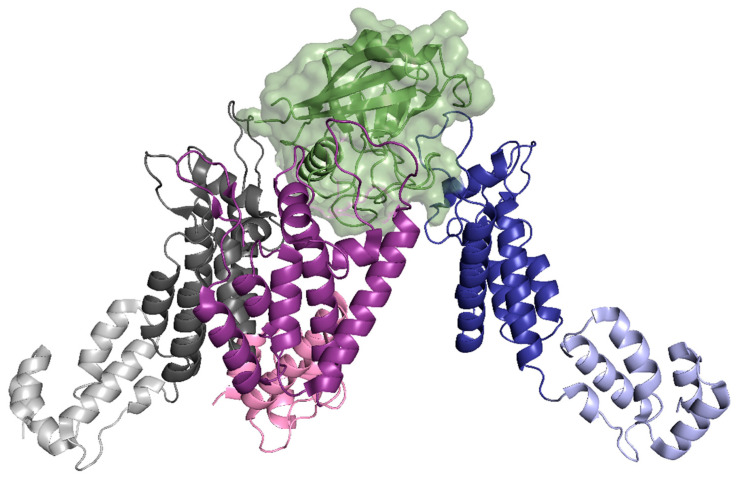
CypA binds to three CA protomers from two adjacent CA hexamers. One protomer of the CA protein is shown with the NTD in dark blue and the CTD in light blue. In total, two protomers from an adjacent CA hexamer are shown with the NTD in dark purple and light grey and the CTD in light pink and light grey. CypA, shown in green, binds to all three protomers through the canonical CypA binding loop (blue protomer) and the non-canonical binding sites (purple and grey). (PDB 6Y9W). Image created with the PyMOL Molecular Graphics System, Version 2.4 Schrödinger, LLC.

A host cell factor implicated in viral uncoating is Cyclophilin A (CypA). It has been determined that CypA is responsible for stabilizing the capsid core and helps facilitate HIV-1 infection [63,64]. CryoEM studies concluded that the CA hexamer is intrinsically curved and that CypA recognizes the curved lattice of the assembled capsid, interacting with three CA protomers from adjacent hexamers (Figure 8) [65]. These interactions include the established CypA binding loop (residues 85 to 93) on the CA-NTD (Figure 8, blue) as well as two non-canonical binding sites (Figure 8, pink and grey) [64,65,66,67]. Through these interactions with the capsid core, CypA is able to block certain restriction factors in human lymphocytes, such as tripartite motif-containing protein 5 alpha (TRIM5α), discussed later in the paper [68,69].

#### 3.2.3. PDZ Domain-Containing Protein 8 and Pin1 have Competing Effects on the Capsid Core

There have been multiple additional host factors identified that stabilize the capsid core. One of these is the PDZ domain-containing protein 8 (PDZD8), which has been shown to interact with both the Gag polyprotein and capsid, resulting in increased infectivity [70,71]. The coiled-coil domain of PDZD8 binds to CA, while other domains of the protein (PDZ domain) are responsible for stabilizing the capsid. In PDZD8 knockdown cells, the rate of capsid disassembly increased while the efficiency of early-stage infection decreased [72]. However, PDZD8-knockout cells were still able to be infected at the same level as PDZD8-expressing cells indicating that PDZD8 is not essential for HIV-1 infection, further demonstrating the adaptability of HIV-1 [70].

An additional host cell factor with a competing effect on viral uncoating is the peptidyl-prolyl cis-trans isomerase NIMA-interacting 1 (Pin1) protein, which induces capsid core uncoating [73]. Suppression of Pin1 resulted in attenuation of HIV-1 replication and an increase in capsid core particulates in the cytosol of the host cell [74]. It has been found that Pin1-CA interactions occur through the phosphorylated Ser16 on CA, which is phosphorylated by the extracellular signal-regulated kinase 2 (ERK2) [75].

#### 3.2.4. ERK2 and MELK

Extracellular Signal-Regulated Kinase 2 (ERK 2) and Maternal Embryonic Leucine Zipper Kinase (MELK) have been implicated in CA function. ERK2 phosphorylates Ser16 on CA [75]. Pharmacological inhibition of ERK activity results in decreased Ser16 phosphorylation and impaired reverse transcription, leading to reduced viral replication [75]. Additionally, genetic suppression of ERK2 expression resulted in attenuated ERK2 packaging inside virions and decreased CA phosphorylation [75]. Additional host cell factors implicated in uncoating include MELK, which is responsible for optimal uncoating and cDNA generation. MELK phosphorylates Ser149 of CA, resulting in proper disassembly of the CA core [76]. MELK deficient cells demonstrated delayed disassembly and inhibition of reverse transcription, while small-molecule MELK inhibitors have been found to reduce the infectivity of HIV-1 [76]. The effects of ERK2 and MELK on HIV-1 infection illustrates the importance of phosphorylation on HIV-1 infectivity.

### 3.3. Cytoplasmic Trafficking and Associated Host Factors

#### 3.3.1. Capsid Facilitates Retrograde Trafficking by Hijacking Cytoskeleton-Associated Proteins

In addition to uncoating, capsid plays a vital role in cytoplasmic trafficking during HIV-1 infection. After receptor-mediated entry of the virus into the host cell, the capsid core is responsible for facilitating movement towards the nucleus (retrograde movement) for nuclear import. Yeast two-hybrid screening indicated that microtubule-associated proteins MAP1A and MAP1S interact with the viral capsid, and depletion of MAP1A/MAP1S leads to drastically attenuated infectivity [77]. The attenuation was due to impaired retrograde trafficking suggesting that MAP1A and MAP1S play a role in connecting the capsid protein to the host cell’s microtubule network [77].

#### 3.3.2. FEZ1 and BICD2 Link Capsid to Motor Proteins

In addition to these microtubule-associated proteins, fasciculation and elongation factor zeta 1 (FEZ1) have also been shown to bind to capsid directly. FEZ1 is a kinesin-1 adaptor protein that directly interacts with the central pore of the CA hexamer, competing with IP6 and dNTPs. Through this interaction with the R18 pore, the adaptor protein promotes the early stages of infection by allowing for net retrograde transport [78,79].

Protein bicaudal D2 (BICD2) is a dynein adapter protein responsible for directly interacting with various dynein cargoes and activating processivity in the dynein-dynein complex. BICD2 has also been implicated in the cytoplasmic trafficking of the HIV-1 core in host cells since it has been found to bind to HIV-1 capsid at the CC3 domain and contribute to the viral complex’s retrograde movement [26,27]. Additionally, the depletion of BICD2 leads to an increased expression of IFN stimulated genes, suggesting this microtubule adaptor protein assists HIV-1 in evading host immune responses [27]. Through these interactions, it can be seen how HIV-1 can hijack various adapter proteins to use kinesin and dynein to move from the cell membrane to the nucleus for nuclear import [80].

The process of uncoating is complex and concurrent with multiple processes of the early stage of HIV-1 infection, including core trafficking. Disruption of microtubules drastically reduces uncoating, while inhibition of either dynein or the kinesin 1 heavy chain (KIF5B) delayed uncoating, providing evidence that uncoating and trafficking may be linked [81].

#### 3.3.3. Capsid can Bind to and Exploit Microtubule Plus-End Tracking Proteins

The RTC can also bind to microtubule plus-end tracking proteins (TIPs), which stabilize microtubules. Cytoplasmic linker-associated protein 2 (CLASP2) is a TIP that capsid utilizes to stabilize microtubules and promote early infection. CLASP2 has a microtubule-binding domain at the N-terminus with a C-terminus CA binding domain, linking cargo to the resultant stabilized microtubule. Upon entry into the cell, the viral core can bind the CLASP2 protein, acting as an adapter to traffic the capsid core to the nucleus [82]. This is significant, as the viral genome cannot be integrated into the host if it does not reach the nucleus.

#### 3.3.4. EB1 and DRFs Coordinate Cytoplasmic Microtubules for Capsid Trafficking

Another protein implicated in early-stage HIV-1 infection is the microtubule plus-end binding protein 1 (EB1), a highly conserved protein that localizes to the spindle and cytoplasmic microtubules, especially at their distal tips. EB1 depletion prevented HIV-1 from inducing stable microtubule structures, suggesting that EB1 can facilitate early HIV-1 infection [83]. Interestingly, while EB1 does not directly bind to the capsid core, it seems to use an adapter protein to interact. The HIV-1 core presents an EB1-like domain through its pentamer interface that can interact with the CLIP170 protein, which is shuttled to the cell periphery by EB1.

In addition to EB1, diaphanous-related formins (DRFs) have been shown to facilitate microtubule stabilization and assist in the intracellular trafficking and timed disassembly of capsid by interacting directly with the capsid core to coordinate these processes [84]. Such findings suggest a mechanism for HIV-1 to highjack host cell machinery to achieve movement in the cell [85].

### 3.4. Nuclear Import and Localization

#### 3.4.1. Capsid and Nucleoporins: the Key to the Nucleus

Along with uncoating and trafficking, capsid plays a crucial role in nuclear import and localization once the core reaches the nuclear pore. Many studies have implicated capsid in interactions with nuclear pore proteins, known as nucleoporins, as well as molecules such as CPSF6 and transportin-1 that are responsible for import into the nucleus and localization to sites of actively transcribed chromatin following import.

The nuclear import of the core begins with docking at the nuclear pore. Docking is mediated through the cytoplasmic filament nucleoporin 358 (NUP358), also called RANBP2, a 358 kDa protein involved in the nuclear import and export of proteins [14,86]. It has been shown that KIF5B causes the re-localization of NUP358 so that it can directly associate with the core through multiple binding surfaces [14]. NUP358 is an isomerase that has been shown to catalyze CA’s cis-trans isomerization and play a role in synchronizing nuclear entry and uncoating [86]. Interestingly, this interaction appears to be dependent on CPSF6 [14].

**Figure 9:**
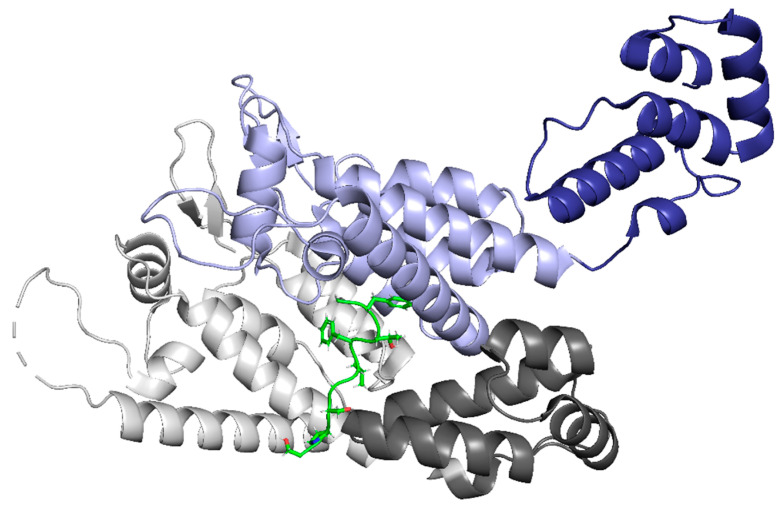
The NUP153 peptide binds between two protomers of the CA hexamer in the NTD-CTD interface pocket. The NTD of protomer A is in light grey, and the CTD is in dark grey. The NTD of protomer B is in light purple, and the CTD is in dark purple. The NUP153 peptide is shown in green. (PDB 5TSX). Image created with the PyMOL Molecular Graphics System, Version 2.4 Schrödinger, LLC.

In addition to the role of NUP358 in nuclear import, nucleoporin 153 (NUP153) is also involved in the import of the capsid core into the nucleus [87]. In NUP153, a phenylalanine-glycine motif interacts with the interprotomer pocket between monomers of the CA hexamer (Figure 9), resulting in the mediated import of the core [88]. It has been shown that mutations that abolish NUP153-CA interactions also inhibit interactions with CPSF6 and result in a core complex that is incapable of nuclear import and infection of non-dividing cells [24]. Additionally, it has been suggested that NUP153 contributes to the integrity of the viral core in the nucleus since the depletion of NUP153 in cells results in increased susceptibility to host cell restriction factors [89]. The N74D mutation in CA has been shown to disrupt the CPSF6-CA interaction and switch the nucleoporin requirement from NUP153 to NUP155 [90]. This indicates that capsid can utilize multiple nuclear import pathways, with the nucleoporins being only one class of protein that the capsid hijacks to achieve nuclear import.

#### 3.4.2. CPSF6 and Transportin-1 Facilitate Nuclear Import and Localization

**Figure 10:**
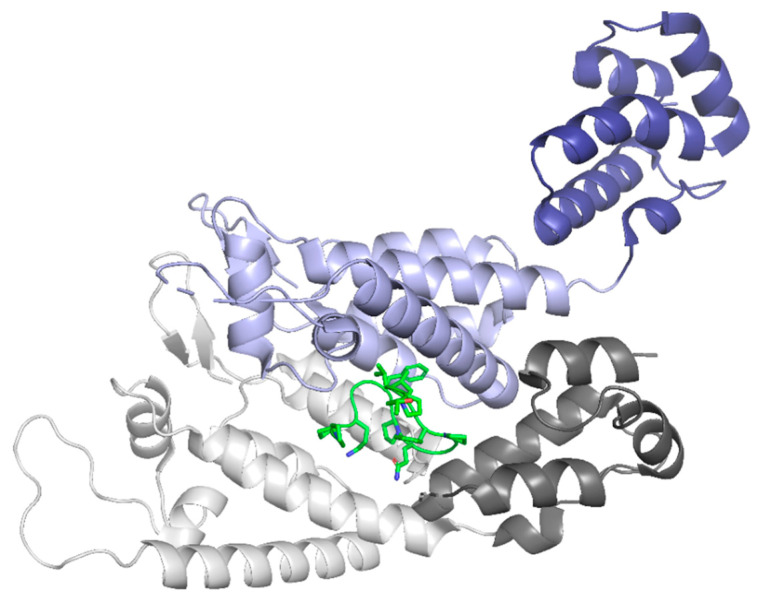
The CPSF6 peptide binds between two protomers of the CA hexamer in the NTD-CTD interface pocket. The NTD of protomer A is in light grey, and the CTD is in dark grey. The NTD of protomer B is in light purple, and the CTD is in dark purple. The CPSF6 peptide is shown in green. (PDB 4WYM). Image created with the PyMOL Molecular Graphics System, Version 2.4 Schrödinger, LLC.

The localization of the PIC inside the nucleus is facilitated by CPSF6 binding within the interprotomer pocket of CA (Figure 10). The interaction between capsid and CPSF6 facilitates nuclear import and nuclear localization, with recent studies showing that CPSF6 drives the PIC towards the nuclear periphery [22,23]. CPSF6 also is involved in localization within the nucleus by associating the PIC with SC35 nuclear speckles [11]. These speckles are highly active transcription sites, suggesting that the CPSF6-CA interaction recruits viral complexes to SC35 speckles to increase reverse transcription [11,91]. When interactions between CA and CPSF6 are halted, PICs accumulate at the nucleus’s edge and uncharacteristically target lamina-associated domains rather than transcriptionally active speckles [92].

Transportin-1 (TRN-1) has been identified as a host cell factor that binds to incoming capsid cores, triggering uncoating and promoting nuclear import [60]. Previous studies have shown that depletion of TRN-1 significantly impedes the early steps of infection [60]. Additionally, TRN-1 promotes the efficient nuclear import of the capsid core and mediates the release of the viral genome from the core [60]. Once imported into the host cell nucleus, these cores, through CPSF6-CA interactions, localize the PIC to SC35 speckles, which are highly active transcriptionally [11].

#### 3.4.3. Transportin-3 Is Crucial for Nuclear Import and Localization

Another important host cell factor that plays a part in the nuclear import for the core of HIV-1 is the transportin-3 protein (TNPO3, also called TRN-SR2). It was initially thought to interact with the HIV-1 integrase protein, although recent studies have suggested that capsid is involved in this interaction [15,16,93,94]. Additionally, it has been determined that TNPO3 plays a role in the integration and nuclear import, as depletion of TNPO3 reduces integration in gene-dense regions [95]. Furthermore, it was found that TNPO3 acts to export viral tRNAs in a RanGTP dependent manner in addition to its capsid interactions. It was proposed that TNPO3 is exploited by HIV-1 and used to displace CA and tRNA in the PIC post-nuclear entry, thereby facilitating integration [94].

### 3.5. Capsid Acts to Shield the Reverse Transcription Machinery from Innate Immune Detection

One question that has been raised is why interferons (IFNs) are not strongly induced through cGAS (cyclic GMP-AMP synthase) sensing in the cytoplasm of the cell during the early stages of HIV-1 infection. The capsid core was hypothesized to act as a shield for the viral nucleic acids by protecting the genome and sequestering the process of reverse transcription, in addition to acting as an essential factor in cytoplasmic trafficking and nuclear import. To investigate this hypothesis, the capsid core was destabilized through genetic mutation and pharmacological inhibition, resulting in decreased infection and increased IFN induction [96]. One HIV-1 variant that has been identified, referred to as the RGDA/Q112D virus, contains five mutations within CA (H87R, A88G, P90D, P93A, and Q112D) that makes it hypersensitive to IFNs [97]. Interestingly, this mutant virus was able to acquire either a Q4R or G94D/G116R mutation in CA, which not only allows for the virus to evade IFN restriction but also results in altered sensitivity to MxB, CPSF6, and CypA and in altered kinetics of reverse transcription and uncoating [97]. These findings indicate that HIV-1 is able to evolve quickly to exploit various pathways to elude IFN-mediated restriction [97].

### 3.6. Gag Lattice Formation and Maturation

The late stage of HIV-1 infection begins after the integration of the viral genome into the host cell genome. This stage includes viral protein production, Gag-lattice formation, the release of an immature virion, and maturation. The HIV-1 CA protein plays an important role in the early stages of the viral life cycle, but its functions are critical in the late stages as well. During assembly, the presence of oligonucleotides has been shown to induce conformational changes in Gag that promote the enthalpically-favorable dimerization of the Gag polyprotein [98]. These Gag oligomers are trafficked to the host cell membrane and begin to form an immature virion. A mechanism for trafficking the Gag polyprotein was demonstrated through interactions with the CA region of Gag and filamin A, a 280 kDa non-muscle actin filament cross-linking protein [99]. Filamin A is known to regulate actin dynamics and is involved in anchoring membrane proteins to the cytoskeleton. Filamin A has also been implicated as a facilitator of cell-to-cell transmission of HIV-1 through binding to and regulating receptor clustering on host cell surfaces. Disruption of the Gag-filamin A interaction eliminates localization and accumulation of Gag at the plasma membrane [99]. Once assembled, the CA and SP1 domains are responsible for stabilizing the Gag-Gag interactions that create the oligomeric lattice of the immature virion [100,101,102,103]. The immature arrangement of CA differs greatly from the mature arrangement of the capsid core. In an immature virion, the CA-CTD plays a central role in stabilizing the immature lattice. Still, after the viral protease cleaves the Gag polyprotein, drastic rearrangements in the CA-NTD occurs, and the CA collapses into the known fullerene cone shape (Figure 4F) characteristic of a mature, infectious virion [102]. This highlights the essential role that CA plays in the late stage of the viral life cycle.

#### IP6 Coordinates CA Assembly

**Figure 11:**
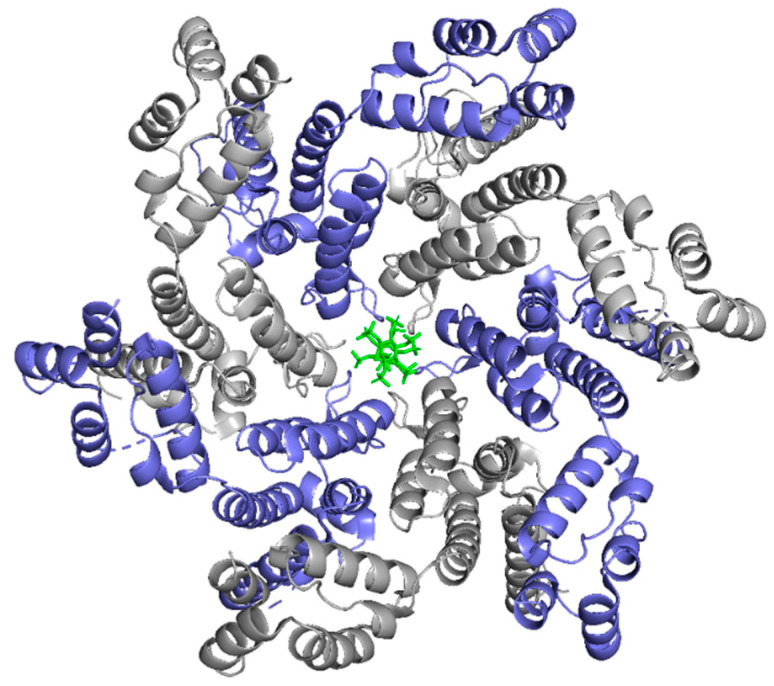
IP6 binds in the R18 pore of the CA Hexamer. Protomers are in alternating colors, slate and grey. IP6 is shown in green interacting with the CA R18 pore. (PDB 6ES8). Image created with the PyMOL Molecular Graphics System, Version 2.4 Schrödinger, LLC.

Inositol hexaphosphate (IP6) is a highly negatively-charged host cell factor present in all mammalian cells [104]. In HIV-1 infection, IP6 has been shown to coordinate capsid assembly and stability [105,106]. The molecule interacts with the positively charged R18 pore at the center of the hexamer structure (Figure 11) [107]. Studies indicate that at clinically relevant levels, IP6 can stabilize the core, limiting spontaneous disassembly and increasing reverse transcription efficiency by promoting the accumulation of DNA within the core [107]. Interestingly, it was shown that IP6 preferentially stabilizes pentamers over hexamers, which was suggested to enhance fullerene cone assembly [108].

A recent study has shown that IP6 stimulates Gag lattice assembly and maturation by facilitating the formation of the six-helix bundle that stabilizes the Gag hexamer [109]. Ablation of IP6 drastically reduced infectious HIV-1 particle production between 10- and 20-fold, highlighting the essential role of IP6 in infection [109]. IP6 interacts with Lys290 and Lys359 of the CA subunit in the Gag hexamer [109]. Upon proteolytic cleavage of Gag, IP6 was found to interact with the capsid and stimulate assembly of the mature capsid lattice [107,109]. Crystallization of mature capsid hexamer in the presence of IP6 illustrated that IP6 binds primarily at the β-hairpins at the N-terminal region of capsid [109].

### 3.7. Interaction with Lipids

Capsid interacts with a wide range of host cell factors but has also been implicated in lipid binding. Previous studies have shown interactions between the CTD of CA and lipid vesicles, in which the CTD undergoes a conformation change upon lipid binding. Such findings further demonstrate CA’s structural flexibility and suggest that CA can bind to the inner leaflet of a virion and coordinate late-stage processes [110].

## 4. Capsid Is a Target of Host Cell Restriction Factors

Host cell restriction factors significantly influence viral infectivity. Host cell restriction factors are proteins in the host cell that can inhibit the viral lifecycle and prevent infection by restricting replication or inducing the innate immune response. In HIV-1, restriction factors can interact with CA to alter its function in various ways, including altering the stability of the core and preventing interactions with host cell factors that facilitate the viral life cycle [111].

### 4.1. MxB Stabilizes the Capsid Core and Represses Nuclear Import

Myxovirus resistance protein B (MxB), a GTPase known to inhibit a variety of viruses, is an alpha-interferon (IFN-α)-inducible restriction factor for HIV-1 infection [112,113]. Recent studies have demonstrated that MxB targets an interface created at the junction of three CA hexamers, leading to increased stabilization of the viral core and subsequent uncoating inhibition [114]. The binding of capsid to MxB is facilitated by MxB dimerization since inhibition of dimerization precludes the binding of MxB to CA [113,115]. In addition to the stabilizing effect of MxB, it appears to repress nuclear import by NUP358 through a CPSF6 dependent pathway [116]. It was also discovered that naturally occurring CA variants might render HIV-1 MxB-resistant while maintaining viral infectivity and interferon induction escape [117].

Interestingly, different clades of HIV-1 seem to be affected by MxB at varying rates. For example, clade C, the dominant HIV-1 clade found in Africa and the most rapidly expanding clade, possesses the most MxB resistant variants [117]. It has been suggested that this supports a potential mechanism where MxB acts as a selective force during HIV-1 evolution [117]. Additionally, it is thought that MxB sensitivity is based on core conformation rather than cofactor recruitment. The binding of CypA induces conformational flexibility, which results in the evasion of restriction factors, such as MxB and TRIM5α [118]. Moreover, the effects of CypA and MxB can be induced or blocked depending on the expression levels of different NUP factors, specifically NUP93, NUP62, NUP88, NUP214, NUP358, and NUP153 [119]. This emphasizes how HIV-1 can exploit different NUP-dependent pathways for nuclear import.

### 4.2. Trim5α Binds to the Capsid Core and Prevents RT

The tripartite motif-containing protein 5α (TRIM5α) is a host cell restriction factor that binds directly to HIV-1 capsid and can restrict infection in non-human primate cells by preventing reverse transcription [120]. Reverse transcription restriction is caused by the oligomerization of TRIM5α into a lattice on the surface of the capsid core. This destabilizes the core, resulting in premature uncoating and inhibited reverse transcription [121]. Interestingly, recent studies have shown that when TRIM5α binds, CA-CypA interactions are abolished. One study showed that by disrupting the CA-CypA interaction, HIV-1 becomes susceptible to TRIM5α. When the CA-CypA interaction is disrupted, TRIM5α associates with the viral core as soon as viral entry occurs, resulting in extreme HIV-1 restriction [69]. Through simulations, binding has been predicted to occur between the SPRY domain of TRIM5α and near the CypA binding loop of the CA, which would account for the abolished interactions between CA and CypA [122,123,124]. TRIM5α binds through its SPRY domain with low affinity (KD > 1mM); however, assembly into a larger oligomeric state, forming a hexagonal lattice, generates enough avidity for sufficient binding [122]. Recent simulations have revealed that TRIM5α initially dimerizes and then diffuses across the capsid core surface to interact with other TRIM5α dimers, which then continue to assemble to form the TRIM5α hexameric lattice that encages the capsid core [122]. Rhesus TRIM5α is the most potent HIV-1 restriction factor known; however, human TRIM5α has lost its ability to restrict HIV-1 and cannot prevent infection [125,126,127,128,129,130].

TRIMCyp is another restriction factor associated with TRIM5 expression and was originally found to restrict HIV-1 in owl monkey cells [131]. TRIMCyp is similar to TRIM5α, but the SPRY domain is replaced with CypA [131]. The CypA region of this fusion protein has been shown to be involved in binding to the capsid core through the CA CypA binding loop since mutation of the CypA binding loop (G89V) and addition of cyclosporin A both can prevent TRIMCyp from properly restricting HIV-1 [132,133,134]. Since the identification of TRIMCyp in owl monkey cells, it has also been identified in other primates highlighting the evolutionary significance of this restriction factor [135,136,137,138].

### 4.3. TRIM34 Restricts Reverse Transcription Independently of IFN Stimulation

The tripartite motif-containing protein 34 (TRIM34), a paralog of the well-known TRIM5α, has also been shown to inhibit HIV-1 infection by targeting the CA protein. TRIM34 restricts reverse transcription independently of interferon stimulation. These inhibitory effects have been proven in CD4+ T cells and monocyte-derived dendritic cells; however, these inhibitory effects are abolished in cells deficient in TRIM5α [139].

### 4.4. Overexpression of TRIM11 Hastens Uncoating

Another TRIM protein that has been implicated in CA binding is TRIM11, a potent inhibitor of HIV-1 through the reduction of viral transcripts. Results show that overexpression of TRIM11 caused increased capsid uncoating and the generation of fewer transcripts, while a knockdown of TRIM11 resulted in increased capsid stability with increased transcript numbers [140].

### 4.5. Daxx Inhibits Capsid Uncoating

A ubiquitously expressed and developmentally essential protein recently implicated in HIV-1 inhibition is the death domain-associated protein 6 (Daxx). Daxx is involved in apoptosis-mediated extrinsic cell death and has also been shown to inhibit reverse transcription in HIV-1 [141]. Daxx acts as a scaffolding protein to regulate the functions of other proteins through interactions between its SIM (SUMO-interacting motif) domain and the target protein [141]. Recent studies have found that Daxx expression is upregulated during HIV-1 infection and suggest that Daxx interacts with CypA-bound capsid and recruits various restriction factors, such as TNPO3, TRIM5α, and TRIM34 [141]. Such interactions with Daxx prevent capsid uncoating, thereby inhibiting reverse transcription and infection [141].

### 4.6. NONO’s Low Affinity Prevents Restriction

Recent studies have found that the non-POU domain-containing octamer-binding protein (NONO), a nuclear RNA and DNA binding protein that serves to activate the cGAS innate immune response pathway, binds to the HIV-1 capsid. However, it has a higher affinity to capsid of the less pathogenic HIV-2. It was concluded that NONO’s low affinity for the HIV-1 capsid is the cause of its inability to restrict HIV-1 infection [142]. Additional studies need to be carried out to determine if this a viable avenue for clinically relevant HIV-1 restriction.

### 4.7. REAF Restriction Is Prevented by Vpr-Induced Degradation

RNA-associated early-stage antiviral factor (REAF) has been reported to interact with HIV-1 during the early stages of the life cycle, affect uncoating and reverse transcription, and inhibit not only HIV-1 but also HIV-2 and SIV. REAF overexpression resulted in fewer detectable infected cells and reverse transcripts. Early studies suggest that REAF associates with viral nucleic acids, inhibiting RT [143]. Mapping of the interaction sites of REAF to MA and CA through mutational studies determined that CA was likely the direct-binding partner. This interaction is a part of the Lv2/REAF mechanism that affects a broad range of lentiviruses [144].

Interestingly, it has been shown that the Vpr viral protein plays a role in preventing REAF from inhibiting viral infection. It was determined that Vpr, within 30 min of core entry into the cytoplasm, induces the degradation of REAF. While REAF levels are upregulated during infection, the presence of Vpr is enough to prevent this response from restricting viral infection [145].

## 5. HIV-1 CA as a Target for Inhibitors

**Figure 12:**
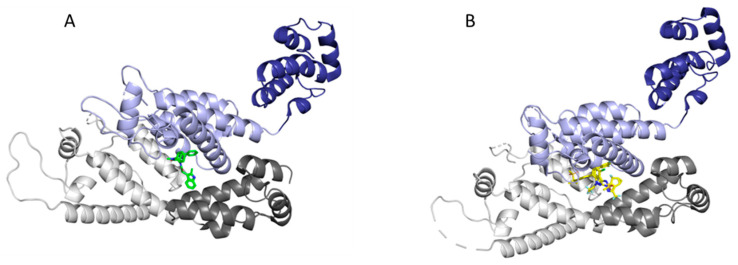
Binding of inhibitors in the interprotomer binding pocket of the CA hexamer. The NTD of protomer A is in light grey and the CTD is in dark grey. The NTD of protomer B is in light purple and the CTD is in dark purple. Inhibitors (**A**) PF74 (PDB 4XFZ) and (**B**) GS-6207 (PDB 6V2F) are shown in green and yellow, respectively. The binding of these inhibitors in the NTD-CTD interface pocket prevents necessary host cell factors CPSF6 and NUP153 from binding to this pocket. Image created with the PyMOL Molecular Graphics System, Version 2.4 Schrödinger, LLC.

Due to the multiple roles and interactions that CA is involved in throughout the HIV-1 lifecycle, as well as its high sequence conservation, CA is an attractive target for therapeutic intervention. Although many CA inhibitors have been discovered, two compounds designed by Pfizer and Gilead are of particular interest due to their potencies and mechanisms of action [33,146,147,148,149,150,151,152,153,154]. One such inhibitor is the Pfizer compound PF-3450074 (PF74), which has been shown to inhibit HIV-1 replication with a potency in the nanomolar range (EC_50_ = 8–640 nM) [155,156]. PF74 binds at the interprotomer pocket (Figure 12A) and has been shown to accelerate the capsid core uncoating, thereby inhibiting reverse transcription in the early stage of the lifecycle [34,154,155,156,157]. Another consequence of PF74 binding in the interprotomer pocket is the displacement of host factors CPSF6 and NUP153, which are necessary interactions for successful nuclear import and integration of the viral contents [42,158,159]. In addition to its inhibitory capabilities in the early stages of the life cycle, PF74 also has an effect on the late stages of the viral life cycle. By increasing the CA multimerization rate, PF74 causes atypical viral morphologies and inhibits the proper maturation of virions [155]. These numerous functions that PF74 has throughout the HIV-1 lifecycle highlight the multimodal mechanism of this CA inhibitor.

Another recently discovered compound that also binds to the CA interprotomer pocket (Figure 12B) is the Gilead compound GS-6207 (Lenacapavir), which has also been shown to have a multimodal mechanism of action with a potency in the low picomolar range (EC_50_ = 55–314 pM) [29,160]. Similar to PF74, GS-6207 functions by stabilizing the capsid core leading to a buildup of intact core in the cytoplasm. GS-6207 prevents adequate binding of CPSF6 and NUP153 to the interprotomer binding site, leading to reduced nuclear import and integration of viral contents [29]. Additionally, GS-6207 demonstrates an inhibitory effect in the late stages of the HIV-1 lifecycle, but the mechanism has yet to be fully elucidated. GS-CA1, the parental compound of GS-6207, was recently found via electron cryotomography and lattice mapping to decrease capsid core integrity and cause substantial disassembly and fractioning of the core [55,161]. It is believed that GS-CA1 stabilizes the CA hexamer causing flattening of the naturally curved capsid, which leads to fracturing of the capsid core. This indicates a potential mechanism by which GS-6207 and other capsid stabilizing inhibitors may also function.

These inhibitors show that exploiting the structure and function of the CA protein is a viable therapeutic avenue to prevent HIV-1 infection and have led numerous research groups to further explore analogs of these compounds with similar binding sites and functional mechanisms [32,33,34,152,153,154,162,163,164,165,166,167].

## 6. Conclusions

The HIV-1 CA is responsible for several crucial processes in the HIV-1 life cycle and is integral in the early and late stages. The 231 residue-long protein forms a wide range of oligomeric conformations, allowing for assembly into the complex fullerene cone structure attributed to a mature virion. It interacts dynamically with various host cell factors involved in cytoplasmic trafficking, such as FEZ1, BICD2, and CLIP170. Capsid is also involved in nuclear import and localization through its interactions with nucleoporins, TNPO-3, and CPSF-6. Capsid is also essential in reverse transcription, where it shields the RTC from host cell restriction factors, such as MxB and TRIM5α. Furthermore, capsid has been implicated in Gag-Gag lattice formation and viral maturation, which are essential stages for the generation of infectious viral particles.

The interactions of capsid and their cellular contexts need to be further investigated as the myriad of host cell factors (Table 1) could indicate different pathways that the virus employs to successfully infect different cells. Exploiting these different pathways and the numerous interfaces that capsid utilizes to interact with them both represent novel avenues to explore for therapeutic development. Although much is left to be elucidated, unveiling the interaction partners of the capsid, the mechanisms of how they interact and function, and the kinetics of these events could provide more opportunities and targets for anti-HIV-1 therapies.

## Figures and Tables

**Table 1:** Summary of capsid-binding host cell factors.

Abbreviation	Name	Binding Site on CA	Related Process	Complex PDB ID
**ATP**	Adenosine triphosphate	R18 pore	Reverse Transcription	-
**BICD2**	Bicaudal D2	-	Trafficking	-
**CLASP2**	Cytoplasmic linker-associated protein 2	-	Trafficking	-
**CLIP170**	Cytoplasmic linker protein 170	EB1-like domain at pentamer interface	Trafficking	-
**CPSF6**	Cleavage and polyadenylation specificity factor 6	Interprotomer Pocket	Nuclear Import and Localization	4WYM
**CypA**	Cyclophilin A/Peptidylprolyl isomerase A	CypA loop (residues 85 to 93)/Inter-hexamer interface	Uncoating	5FJB 6Y9W 6Y9V
**Daxx**	Death domain-associated protein	CA-CypA complex	Uncoating Inhibition	-
**dNTP**	Deoxynucleoside triphosphate	R18 pore	Reverse Transcription	-
**DRFs**	Diaphanous-related formins	-	Uncoating/Trafficking	-
**ERK2**	Extracellular signal-regulated kinase 2	Phosphorylates Ser-16 of CA	Uncoating	-
**FEZ1**	Fasciculation and elongation protein zeta-1	R18 pore	Trafficking	-
**IP6**	Inositol Hexaphosphate	R18 pore	Reverse Transcription	6ES8 6BHT
**MAP1A/MAP1S**	Microtubule-associated proteins 1A and 1S	Monomeric CA interface	Trafficking	-
**MELK**	maternal embryonic leucine zipper kinase	Phosphorylates Ser-149 of CA	Uncoating	-
**MxB**	Myxovirus resistance protein B	Inter-hexamer interface	Uncoating Inhibition/Nuclear Import Inhibitor	-
**NONO**	Non-POU domain-containing octamer-binding protein	CA NTD	cGAS Response	-
**NUP153**	Nucleoporin 153	Interprotomer Pocket	Nuclear Import	4U0C 6AYA
**NUP358**	Nucleoporin 358	CA NTD	Nuclear Import	4LQW
**PDZD8**	PDZ domain-containing protein 8	Gag	Uncoating	-
**Pin1**	Peptidyl-prolyl cis-trans isomerase NIMA-interacting 1	Phosphorylated Ser-16 of CA	Uncoating	-
**REAF**	RNA-associated early-stage antiviral factor	-	Uncoating Inhibitor/Reverse Transcription Inhibitor	-
**TNPO3/** **TRN-SR2**	Transportin 3	CA-CPSF6 complex	Uncoating, Nuclear Import and Integration	-
**TRIM11**	Tripartite motif-containing protein 11	-	Uncoating	-
**TRIM34**	Tripartite motif-containing protein 34	-	Reverse Transcription Inhibition	-
**TRIM5α**	Tripartite motif-containing protein 5	Capsid lattice near CypA binding loop	Uncoating Inhibition	-
**TRN1**	Transportin 1	CypA binding loop (G89 crucial)	Nuclear Import	-

## Data Availability

No new data were created or analyzed in this study. Data sharing is not applicable to this article.
